# Structure of an inactive RNA polymerase II dimer

**DOI:** 10.1093/nar/gkab783

**Published:** 2021-09-16

**Authors:** Shintaro Aibara, Christian Dienemann, Patrick Cramer

**Affiliations:** Department of Molecular Biology, Max Planck Institute for Biophysical Chemistry, Am Fassberg 11, 37077, Göttingen, Germany; Department of Molecular Biology, Max Planck Institute for Biophysical Chemistry, Am Fassberg 11, 37077, Göttingen, Germany; Department of Molecular Biology, Max Planck Institute for Biophysical Chemistry, Am Fassberg 11, 37077, Göttingen, Germany

## Abstract

Eukaryotic gene transcription is carried out by three RNA polymerases: Pol I, Pol II and Pol III. Although it has long been known that Pol I can form homodimers, it is unclear whether and how the two other RNA polymerases dimerize. Here we present the cryo-electron microscopy (cryo-EM) structure of a mammalian Pol II dimer at 3.5 Å resolution. The structure differs from the Pol I dimer and reveals that one Pol II copy uses its RPB4-RPB7 stalk to penetrate the active centre cleft of the other copy, and vice versa, giving rise to a molecular handshake. The polymerase clamp domain is displaced and mobile, and the RPB7 oligonucleotide-binding fold mimics the DNA–RNA hybrid that occupies the cleft during active transcription. The Pol II dimer is incompatible with nucleic acid binding as required for transcription and may represent an inactive storage form of the polymerase.

## INTRODUCTION

Dimerization of RNA polymerase I (Pol I) has emerged as a mechanism to repress the synthesis of ribosomal RNA (1). Pol I dimers can be isolated from *Saccharomyces cerevisiae* extracts and are inactive in transcription (2). Pol I dimerization was initially observed by electron microscopy at low resolution (3) and later in high-resolution crystal structures (4,5). More recently, a higher resolution structure of the *Schizosaccharomycespombe* Pol I dimer was determined by cryo-EM (6). Structural studies indicated that the Pol I dimer represents a repressed state of the enzyme, leading to a model for Pol I regulation (5). The Pol I dimer is formed by mutual interactions of the active centre cleft of one polymerase copy with the ‘stalk’ subcomplex A14–A43 of the other copy (4,5). In cells, nutrient deprivation or inhibition of either ribosome biogenesis or protein synthesis leads to Pol I dimerization and repression (7). There is evidence that the Pol I stalk and the Pol I-specific A43 ‘connector’ element are involved in dimerization and transcriptional regulation (5,7). The A43 connector invades the cleft of the adjacent polymerase and contacts the clamp and lid loop (4,5). The Pol I dimers retain some flexibility (8) but cannot be resolved when the Pol I initiation factor Rrn3 is added (9), although Rrn3 binding is predicted to interfere with Pol I dimerization (10). Thus, dimerization of Pol I is a regulatory mechanism that has been studied in structural detail.

With respect to RNA polymerase II (Pol II), the yeast enzyme was observed to dimerize within a two-dimensional crystal lattice (11). The Pol II dimer in such a lattice differs from Pol I dimers and is formed via a minor contact between the exterior surface of the enzyme in regions around subunits RPB3 and RPB11. Dimers of yeast Pol II were also observed in three-dimensional crystals (12), but the dimer interface was apparently formed by crystal contacts because it was strongly altered after crystal dehydration and shrinkage (13). Bovine Pol II was also observed in a dimeric state in negative-stain electron microscopy studies in the absence of subunit GDOWN1, but a 3D reconstruction could not be generated due to limitations of the data (14). Imaging studies in yeast were inconclusive with respect to Pol II dimerization (7). Thus, it remains unclear whether Pol II dimerizes in solution, and detailed structures of Pol II dimers have not been reported to date.

Here we present the cryo-EM structure of the Pol II dimer at a nominal resolution of 3.5 Å. The structure reveals a highly defined Pol II-Pol II dimerization interface involving the RPB4-RPB7 stalk and active center cleft. Superposition of structures of functional Pol II complexes predicts that the dimer is incompatible with transcription initiation and elongation. Finally, the Pol II dimer deviates substantially from the known Pol I dimers, although a penetration of the partially conserved stalk subcomplexes into the polymerase cleft is a feature of polymerase dimerization in both cases.

## MATERIALS AND METHODS

### Sample preparation

Pol II from *Sus scrofa domesticus* or *S. cerevisiae* was purified as described (15–18) (Supplementary Figure S1). Each respective sample of polymerase was diluted 5-fold with 10 mM HEPES/NaOH pH 8.0, 200 mM NaCl, 0.45 mM TCEP and 0.01% β-DDM (or without β-DDM for control), resulting in a final concentration of 0.008% β-DDM in the sample used for cryo-EM. Exactly 3 μl of sample was applied on to a freshly glow-discharged holey carbon grid (Quantifoil R2/1 Cu) and incubated for 10 s at 4°C, 100% humidity in a Vitrobot Mk IV system (FEI). The grids were blotted for 3 s prior to plunge cooling in liquid ethane.

### Structure determination

Screening was conducted initially on a Glacios transmission-electron microscope (Thermo Fisher) operated at 200 kV and equipped with a Falcon-III direct-electron detector (Thermo Fisher). The final dataset was acquired on a Titan Krios (Thermo Fisher) operated at 300 kV and equipped with a K3 detector and Quantum LS energy filter with a slit width of 20 eV. Movies were acquired in one continuous session in an automated manner using SerialEM (19) at 105 000× nominal magnification, yielding a pixel size of 0.817 Å. Exposures of 1.491 s were taken resulting in a total dose of 41.97 electrons/Å^2^, with defoci values ranging from –0.2 to -4 μm. A total of 16 133 movies were acquired over 3 days and were aligned and averaged using patched movement correction in Warp (20). Contrast transfer function (CTF) parameters were estimated using GCTF (21) and particles were picked within Warp using the standard Box2NetMask_20180918 network. All subsequent image-processing steps were performed in RELION-3.0.7 (22) (Supplementary Figure S2).

From the 16 133 micrographs, 886 094 particles were extracted with a binning factor of 5. These particles were subjected to multiple rounds of reference-free 2D classification to discard poorly aligning particles. At this point, the dataset was crudely divided into sets of dimer-like (410 841 particles) and monomer-like particles (211 320 particles). Candidate dimeric particles were re-extracted without binning and subjected to a consensus 3D auto-refinement with two-fold rotational symmetry applied (C2) to align the particles on to a single reference, the first of which was generated *de novo* using the stochastic gradient descent approach within RELION (23). The particles were then subjected to one round of CTF refinement and Bayesian polishing to improve the signal of the particles for the subsequent classification steps. After the polishing procedure, the particles were re-aligned with global sampling but this time without enforcing any symmetry (C1). Using these angles as starting points, two-rounds of 3D classification with local angular sampling (C1) were conducted to remove any contaminating monomeric Pol II as a first step, and then identifying the most promising dimeric classes as a second. After these two rounds of 3D classification, 218 750 particles were retained and subjected to a consensus 3D auto-refinement with C2 symmetry imposed. Afterward, 3D classification with local angular sampling (C2) was conducted to identify distinct states of the dimeric Pol II, of which three classes could be selected. Each individual class was subjected to then another round of polishing (i.e. CTF refinement and Bayesian polishing sandwiched by 3D auto-refinements) to improve the resolution, and this yielded three states of the dimeric Pol II with resolution of 3.5 Å (class 1), 3.8 Å (class 2) and 3.8 Å (class 3) based on the FSC = 0.143 criterion (Supplementary Figure S3). 3D-FSCs were calculated using the remote 3DFSC processing server (24).

To improve the local resolution of Pol II, symmetry was allowed to relax by means of symmetry expansion (25). Each particle was duplicated and rotated about the 2-fold rotation axis and allowed to align locally to one copy of Pol II that was signal subtracted. This procedure improved some regions of Pol II and was used to assist model building. The relaxation of symmetry indicated that there was unresolved heterogeneity within the classes and improved the resolution of each class somewhat: 3.1 Å (class 1), 3.4 Å (class 2) and 3.5 Å (class 3).

For candidate monomeric Pol II particles, classes showing robust density with the stalk of Pol II visible were selected using 3D classification with local angular sampling. To establish the angles, particles were re-extracted without binning and consensus refinement using a reference that was also generated *de novo* using the stochastic gradient descent approach was done. After three rounds of 3D classification, 92 854 particles that corresponded to monomeric Pol II were identified that generated a reconstruction with an overall resolution of 3.1 Å.

Atomic models were built using Coot (26) starting from the transcribing Pol II (PDB ID: 5FLM) as an initial starting model. Atoms corresponding to nucleic acid and the RPB1/RPB2 clamp module were deleted, and the remaining residues were adjusted into the map on a residue-by-residue basis. Restrained refinement was performed using PHENIX 1.17 (27) where strict NCS constraints were enforced to keep the two Pol II copies identical as the map has imposed C2 symmetry. The final models were validated using MolProbity (28), with the final statistics given in Table 1.

**Table 1.** Cryo-EM data collection and processing statistics

Data collection and pre-processing				
Magnification	105 000×			
Voltage (kV)	300			
Electron exposure (e–/Å^2^)	41.97			
Defocus range (μm)	0.2–4			
Pixel size (Å)	0.817			
Initial particle images (no.)	886 094			
	Dimer Class 1	Dimer Class 2	Dimer Class 3	Monomer
EMDB accession code	EMD-13129	EMD-13130	EMD-13131	EMD-13132
Symmetry imposed	C2	C2	C2	C1
Final particle images (no.)	71 781	47 091	41 379	92 854
Map resolution (Å) FSC threshold = 0.143	3.5	3.8	3.8	3.1
Map resolution range (Å)	3.2–5.6	3.5–7.1	3.5–6.7	2.9–7.7
Map sharpening *B* factor (Å^2^)	-77	-93	-92	-39
**Refinement**				
PDB accession code	7OZN	7OZO	7OZP	
Initial model used (PDB code)	5FLM	7OZN	7OZN	
Model resolution (Å) FSC threshold = 0.5	3.7	4.1	4.2	
Model composition				
Non-hydrogen atoms	54 330	54 330	54 330	
Protein residues	6774	6774	6774	
Ligands	Zn: 10	Zn: 10	Zn: 10	
Mean *B* factors (Å^2^)				
Protein	51.00	76.54	61.19	
Ligand	121.57	149.17	151.76	
R.m.s. deviations				
Bond lengths (Å)	0.002	0.002	0.002	
Bond angles (°)	0.416	0.380	0.380	
Validation				
MolProbity score	1.24	1.27	1.27	
Clashscore	4.64	4.59	4.68	
Poor rotamers (%)	0.00	0.00	0.00	
Ramachandran plot				
Favored (%)	98.01	97.86	97.89	
Allowed (%)	1.99	2.14	2.11	
Disallowed (%)	0.00	0.00	0.00	

## RESULTS

### Structure of the Pol II dimer

Dimers of Pol II are often observed as a minor population across different Pol II-containing preparations used for cryo-EM studies (29,30). These dimers are generally discarded early during data processing as they often represent substantially <1% of the dataset. As a result of this, the scarcity of dimeric Pol II species has precluded their structural characterization. We were however able to enrich for the dimeric form of *S. scrofa domesticus* Pol II in solution by the addition of the non-ionic detergent *n*-dodecyl β-D-maltoside (DDM, Materials and Methods), which is typically used in the solubilisation of membrane proteins and for improving orientation distribution of proteins in cryo-EM (31,32). No additional reagents, such as cross-linkers or other fixatives, were required to enrich for Pol II dimers. The addition of DDM at a final concentration of 0.008% (w/v) increased the amount of Pol II dimers observed on the grid and enabled determination of the structure to 3.5 Å resolution with C2 symmetry enforced (Supplementary Figures S2 and S3).

**Figure 1.**
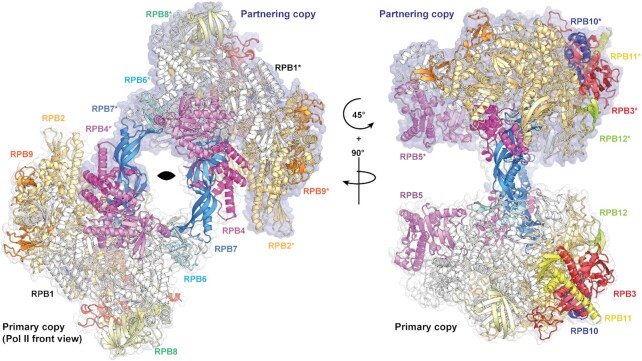
Structure of Pol II dimer. Overall view of the atomic model of the Pol II dimer (class 1) when viewed from the front of the primary copy (bottom). Each of the chains are colored by their canonical colors and the chains belonging to the partnering copy of the dimer are denoted with asterisks. The two-fold rotation axis is indicated in the left image, and surface representations (white and blue) indicate the two monomers.

The Pol II dimer structure contains two copies of each of the 12 polypeptide chains found in a transcription-competent Pol II (RPB1-12) (33). The dimer is formed through a handshake-like interaction between the stalk of one Pol II copy and the active centre cleft of the other copy (Figure 1 and Supplementary Movie S1). In the resulting 2-fold symmetric arrangement, the two stalks are arranged near the centre of the complex with the cores of the two Pol II copies on the periphery. There was no cryo-EM density for the clamp domains in any of our reconstructions and no significant protein degradation was observed for either RPB1 or RPB2 by SDS-PAGE (Supplementary Figure S1A), indicating that the clamp is mobile. A mobile clamp is typical for preparations of mammalian Pol II that do not contain nucleic acid (34) and also occurs in a minor population of the Pol II elongation complex (EC) (35) and the pre-initiation complex (PIC) (30). Analysis of where the regions of disorder commences shows that the ‘switch regions’ (36) in RPB1 and RPB2 demarcate the boundaries between robust and diffuse density (Supplementary Figure S4). Therefore, it is likely that the switch regions play a role in clamp opening for the mammalian Pol II. We also observed dimers for Pol II from the yeast *S. cerevisiae*, which has a less mobile clamp (Supplementary Figure S1B and C), but due to preferential orientation bias were unable to reconstruct a 3D volume. We therefore suggest that a high mobility of the clamp is not a prerequisite for Pol II dimerization but may play a role in stable dimer formation.

### Pol II–Pol II interactions

**Figure 2.**
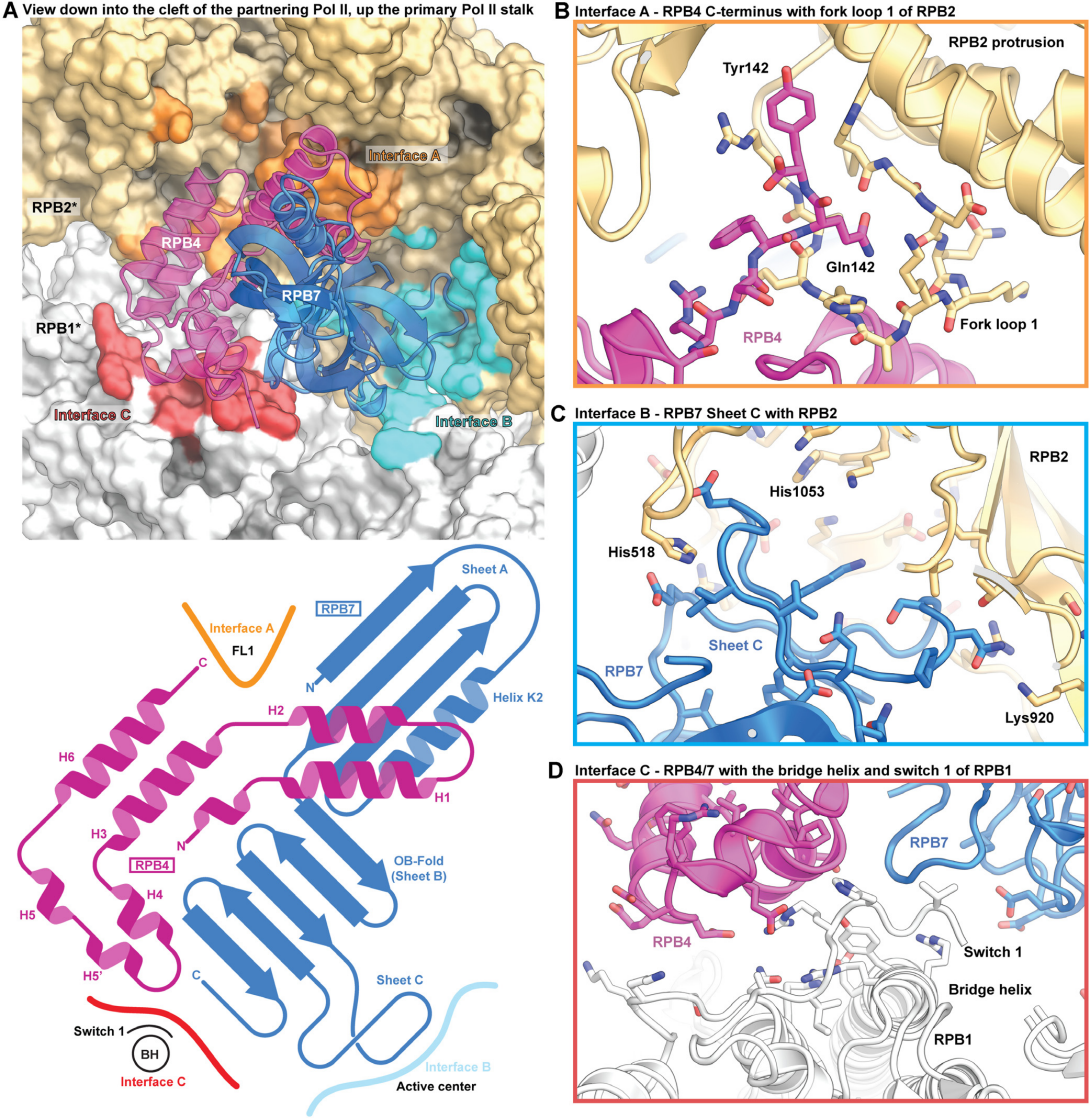
Pol II–Pol II dimer interface. (**A**) Dimerization interface of Pol II–Pol II homodimer; the RPB4/RPB7 stalk of the partnering Pol II copy occupies a large portion of the Pol II cleft and interacts with three key areas of the Pol II core (interfaces A, B and C). A schematic domain representation is shown below that depicts the secondary structural regions of the RPB4/RPB7 stalk that interface with the partnering Pol II monomer. (**B**) Interface A involves Pol II elements fork loop 1 and fork loop 2. (**C**) Interface B involves interaction of the OB-fold and sheet C of RPB7 with the DNA-RNA hybrid-binding cleft, and (**D**) interface C includes the switch 1 region and the bridge helix.

The Pol II–Pol II dimer interface is formed through an intimate interaction between the RPB4-RPB7 stalk of one Pol II copy and numerous elements of the core of the other Pol II copy, formed by RPB1 and RPB2 (Figure 2A and Supplementary Figure S2). The stalk nestles deeply in the active center cleft of the other Pol II copy and buries ∼2100 Å^2^ of surface area per copy. Due to these extended interactions, the stalk displayed very good cryo-EM density, allowing us to trace the entire chain of RPB4 until the C-terminal residue Tyr142 (Supplementary Figure S5a).

The residues in the dimer interface cluster in three areas of the Pol II core that we term interfaces A, B and C. Interface A is formed by specific interactions between RPB4 of the primary copy with fork loops 1 and 2 (FL1 and FL2) of RPB2 in the partnering copy (Figure 2B). Tyr142 of RPB4 was wedged between the RPB2 protrusion and lobe domain and Gln141 stabilizes FL1 in a distinct conformation that differs from those observed in the EC or the PIC. Residues 128–137 in helix 6 of RPB4 were also in close proximity to FL2 that has been implicated in DNA bubble maintenance. Here we observed that FL2 adopts a compact helical conformation that differed from that observed in the EC (Supplementary Figure S5B). This conformation however is similar to that seen in the recent high-resolution structures of the mammalian PIC with closed DNA (30).

With respect to the two other interfaces, interface B is formed between RPB7 in one copy and the site that binds the DNA–RNA hybrid in an EC, including the active site of the other Pol II copy (Figure 2C). The oligonucleotide-binding (OB) domain of RPB7 is rich in acidic residues and sheet C (residues 115–137) in particular reaches the active site, perhaps mimicking nucleic acid phosphates. The active site was partially flexible and thus the aspartate loop and the associated catalytic metal ion A were not observed in the reconstructions. However, this was not a consequence of dimerization as the classes corresponding to monomeric Pol II without nucleic acid also displayed similar density in this region (Supplementary Figure S5C). This may indicate that in the absence of nucleic acids or under these particular buffer conditions, the active site region of mammalian Pol II is more mobile than during active transcription. Finally, interface C is formed by interactions of RPB4 and RPB7 with RPB1 residues 1417–1419 that are located in the switch 1 region that connects the Pol II core to the mobile clamp (Figure 2D). We also observe that RPB4 and RPB7 both interact with residues of the bridge helix that spans the active center cleft. The switch 1 is involved in positioning the mobile clamp and differs in conformation between the EC and the PIC (Supplementary Figure S5C).

### The Pol II dimer is predicted to be inactive

**Figure 3.**
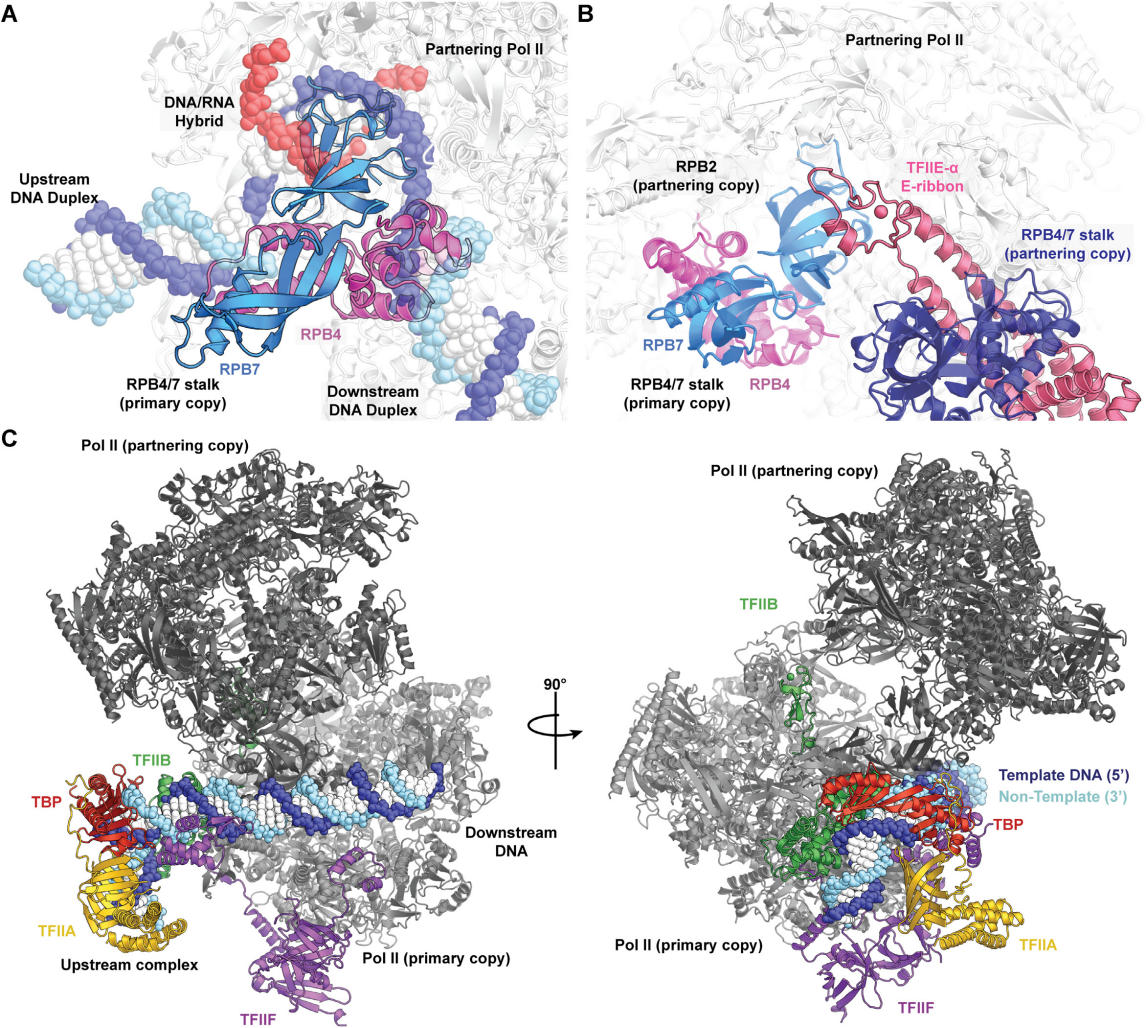
The Pol II dimer is incompatible with transcription activity. (**A**) Superposition of the DNA–RNA hybrid from an active Pol II elongation complex (35) (PDB ID: 5FLM) shows that RPB4 clashes with both the upstream and downstream DNA duplexes, whereas the OB-fold of RPB7 occupies the DNA/RNA hybrid binding site. (**B**) Superposition of the PIC (30) leads to a steric clash between the partnering RPB4-RPB7 stalk and the bulk of TFIIE, although the E-ribbon domain can in principle be accommodated. (**C**) Other early general transcription factors (TFIIA, TFIIB, TBP and TFIIF) and closed promoter DNA are in principle compatible with the dimeric Pol II.

The structure of the Pol II dimer is incompatible with transcription activity. As mentioned above, the RPB4-RPB7 stalk is placed in areas of the Pol II cleft that are critical for activity. Superposition of the Pol II EC structure containing a closed clamp (35) on to the Pol II core of the dimer led to clashes of the closed clamp with the stalk of the partnering Pol II copy in the dimer, showing that Pol II dimerization requires opening of the clamp. Furthermore, RPB4 sterically clashes with the incoming downstream DNA, the OB fold of RPB7 clashes with the DNA–RNA hybrid, and the tip domain of RPB7 clashes with the upstream DNA duplex (Figure 3A). Thus, Pol II dimerization clearly precludes binding of the DNA–RNA hybrid to the Pol II active center and formation of an EC.

Structural superposition of the pre-initiation complex (PIC) (30) reveals that the TATA box binding protein (TBP) and the other general transcription factors TFIIA, -B, -F and –H are in principle compatible with the Pol II dimer, but TFIIE clashes with the RPB4-RPB7 stalk of the partnering Pol II copy (Figure 3B and C). Binding of the general coactivator for initiation, Mediator, is also incompatible with the dimer because Mediator binds the Pol II stalk (37–39). Promoter DNA may also in principle engage with one Pol II copy as closed DNA duplex, but hypothetical DNA opening leads to a clash with the stalk of the other Pol II copy. In summary, the dimer structure is in principle compatible with initial promoter DNA association, but incompatible with promoter opening, initiation and elongation, which are all required for transcription activity.

### Comparison with Pol I dimer structures

**Figure 4.**
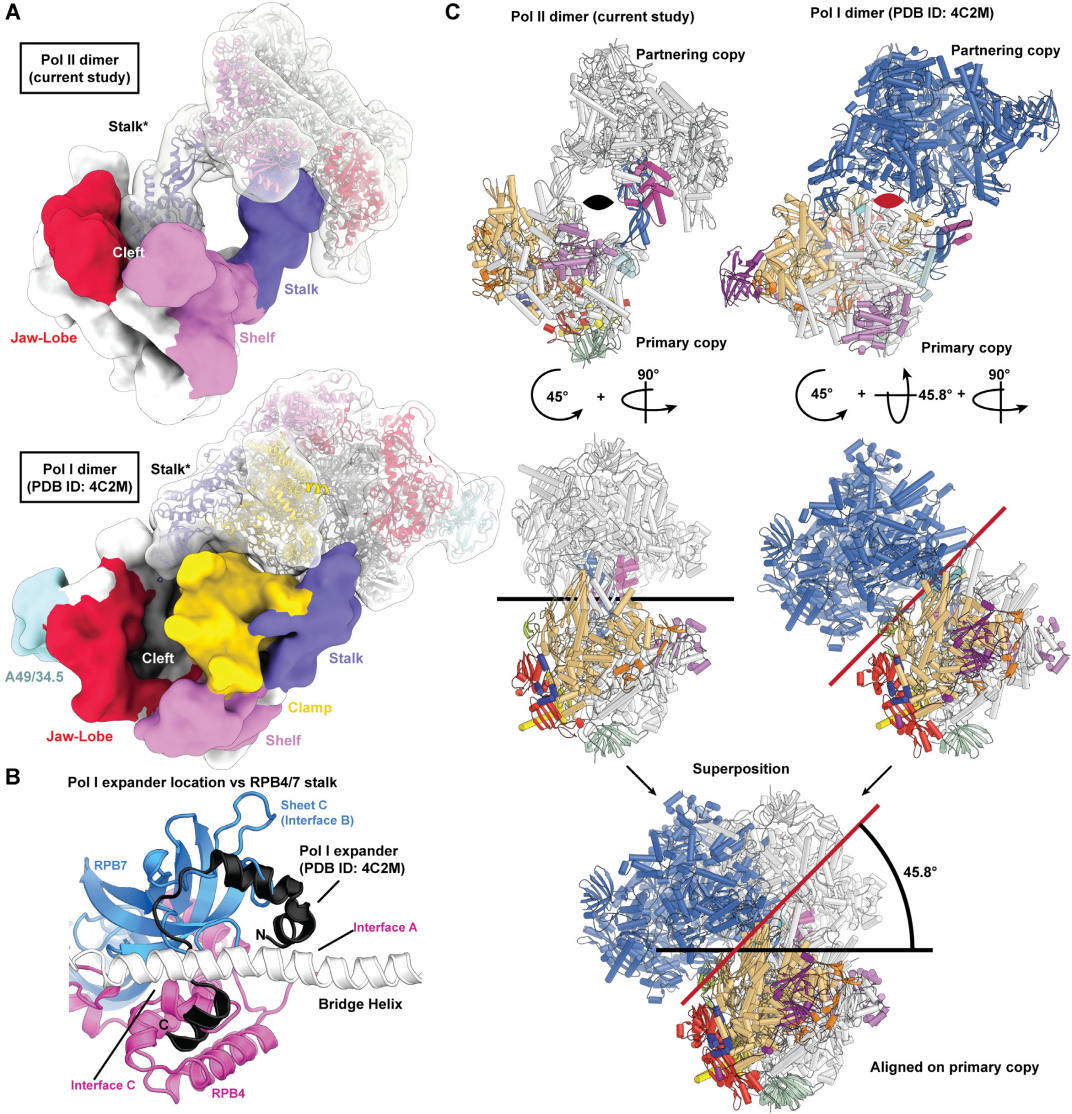
Comparison of Pol II dimer with Pol I dimer structures. (**A**) Different modes of dimer formation. The two different polymerase dimers are depicted as a low-pass filtered surface and coloured according to mobile modules. (**B**) Comparison of the expander loop in Pol I (PDB ID: 4C2M) and the contacts formed in the Pol II dimer. (**C**) Superposition of the Pol I dimer (5) demonstrates that when aligned with a single copy of polymerase, the two-fold rotation axis (depicted either as a black or red line) are tilted ∼45 degrees with respect to the Pol II dimer structure. Compared to the alternative Pol I dimer structure an additional tilt of 7.5 degrees is required.

Comparison of our structure with the *S. cerevisiae* Pol I dimer structure shows that for either dimer, the stalk of one copy of the polymerases reaches toward the partnering copy (Figure 4A). However, due to the flexible clamp in the mammalian Pol II dimer, the Pol II stalk penetrates further into the cleft, whereas the Pol I dimer retains a rigid clamp, preventing such penetration. The stalk occupies a similar area where the Pol I specific ‘expander loop’ (4,5) was observed (Figure 4B). As a result of this hand-shake, the Pol II copies are closer to each other whereas the Pol I copies are further apart from each other. Although both polymerase dimers are related by a two-fold axis (C2 axis), the C2 axis of the two dimers are rotated by ∼45 degrees with respect to each other (Figure 4C). The exact location of the C2 axis can vary because the Pol I dimers show some flexibility with respect to their relative orientation (6,8). In our dataset, we could also classify two more distinct states of the Pol II dimer that display a rotation of one Pol II copy with respect to another, leading to slightly different locations of the C2 axis (Supplementary Figure S6 and Supplementary Movie S2). In summary, the dimers of Pol I and Pol II differ in their exact structural configuration, but in both cases dimerization involves the polymerase stalks.

## DISCUSSION

In the past it has been difficult to interpret the relevance of higher order assemblies of macromolecular structures determined by X-ray crystallography due to potential artifacts introduced by crystal packing. This also relates to the relevance of putative Pol II dimers that had been observed in 2- and 3-dimensional crystals. To overcome these issues, we here used cryo-EM to study Pol II dimerization in solution. We found that the addition of low concentrations of a detergent allowed for structure determination of a Pol II dimer that is also present in solution under non-detergent conditions, although usually as a minor fraction. Comparison of the Pol II dimer with structures of monomeric Pol II in functional complexes showed that Pol II could engage neither with nucleic acids nor with several critical initiation and elongation factors, indicating that the dimer is inactive. Our model differs from the previous observation of a bovine Pol II dimer (14), showing a high mobility of the clamp that enables the stalks of the Pol II monomers to bind to the clefts of the partnering enzymes. However, the 2D class averages of the bovine Pol II dimer are similar to the ones observed in our study and thus this dimeric form of Pol II may be conserved across mammals.

Enzyme inactivation through dimerization is a biological mechanism that is often used for functional repression or storage. For example, bacterial 70S ribosomes dimerize under nutrient-starved conditions with the help of a factor that blocks the mRNA-binding channel (40,41). Pol I can also form an inactive dimeric state under stress conditions such as nutrient starvation (7). The possible temporary inactivation of Pol II through dimerization has however not been described. Nevertheless, Pol II is known to form clusters in the cell nucleus that are observed as sharp foci by super-resolution imaging (42,43) and likely exhibit a very high local Pol II concentration. It is plausible that the inactive Pol II dimer described here may be enriched in such clusters (42). In this respect it is intriguing that binding of the TATA-box binding protein (TBP) and the general transcription factors TFIIA, TFIIB, and TFIIF together with promoter DNA to one Pol II copy of the dimer may in principle be possible, but that TFIIE and Mediator show large clashes with the second Pol II copy in the dimer, and so may play a role in dissociating the dimer for Pol II initiation.

## DATA AVAILABILITY

Atomic coordinates for the reported cryo-EM structures have been deposited with the Protein Data Bank under accession number 7OZN (Class 1), 7OZO (Class 2) and 7OZP (Class 3). Cryo-EM reconstructions for the reported structures have been deposited with the Electron Microscopy Database under the accession number 13129 (Class 1), 13130 (Class 2), 13131 (Class 3) and 13132 (Monomer).

## Supplementary Material

gkab783_Supplemental_Files
