# Supplementary material for: Structure of an inactive RNA polymerase II dimer
Source: Nucleic Acids Res. 2021 Sep 16;49(18):10747–55. doi: 10.1093/nar/gkab783 (PMC8501987; doi:10.1093/nar/gkab783)
Supplement: gkab783_Supplemental_Files [file gkab783_supplemental_files.zip › Aibara_Dimer_210819_supplementary.pdf]

SUPPLEMENTARY DATA

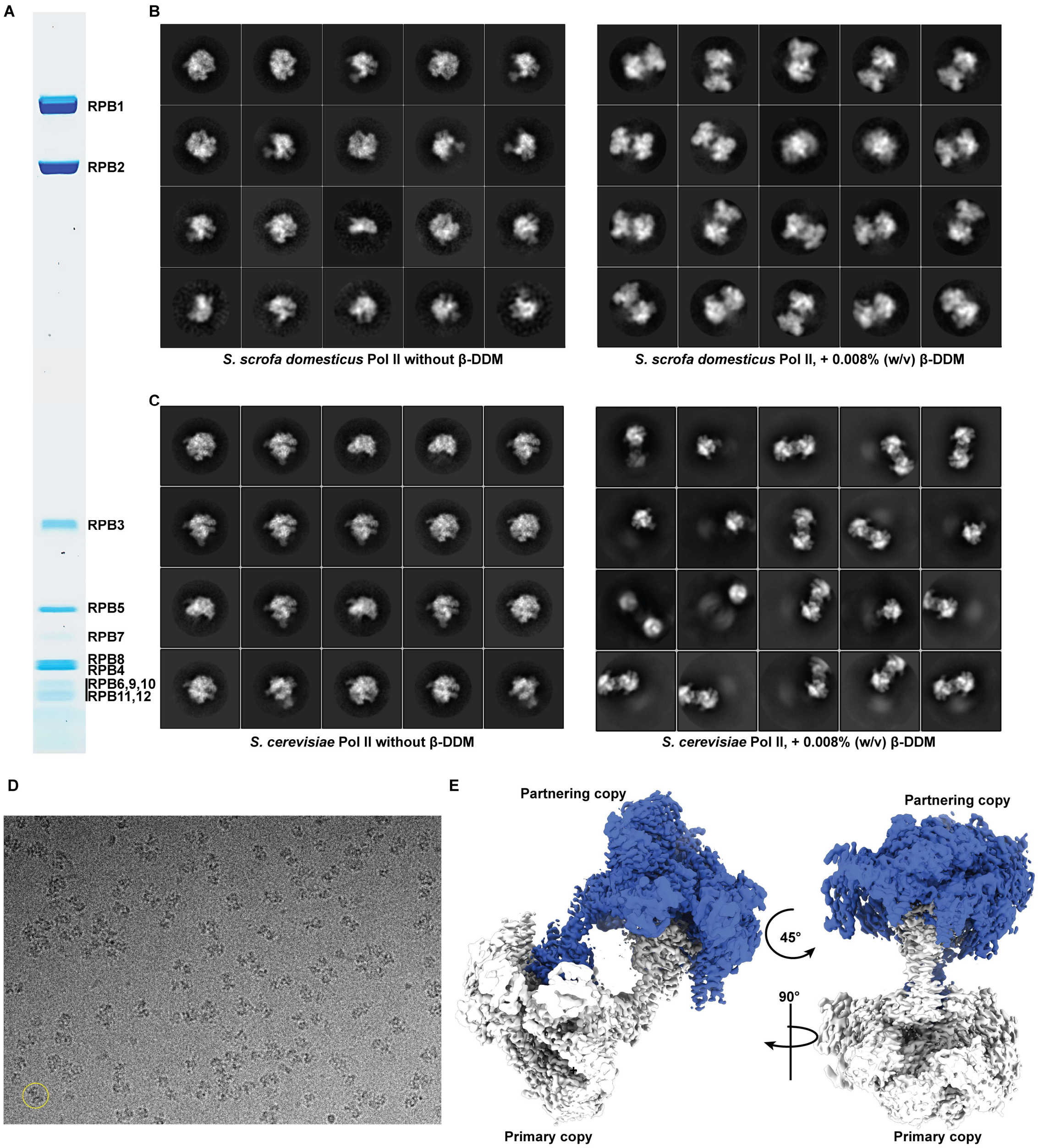

Supplementary Figure 1. Cryo-EM analysis. (A) SDS-PAGE analysis of the Pol II sample used in this study. There is no visible degradation of either RPB1 or RPB2, indicating that there is unlikely to be proteolytic degradation of the clamp. **(B)** Top 20 2D classes from screening datasets demonstrating the effect of the addition/omission of β-DDM to *S. scrofa domesticus* Pol II and **(C)** *S. cerevisiae* Pol II. **(D)** Representative cryo-EM micrograph, circle represents 280 Å. **(E)** Cryo-EM map of class 1, viewed as the same in Figure 1, coloured by each Pol II monomer.

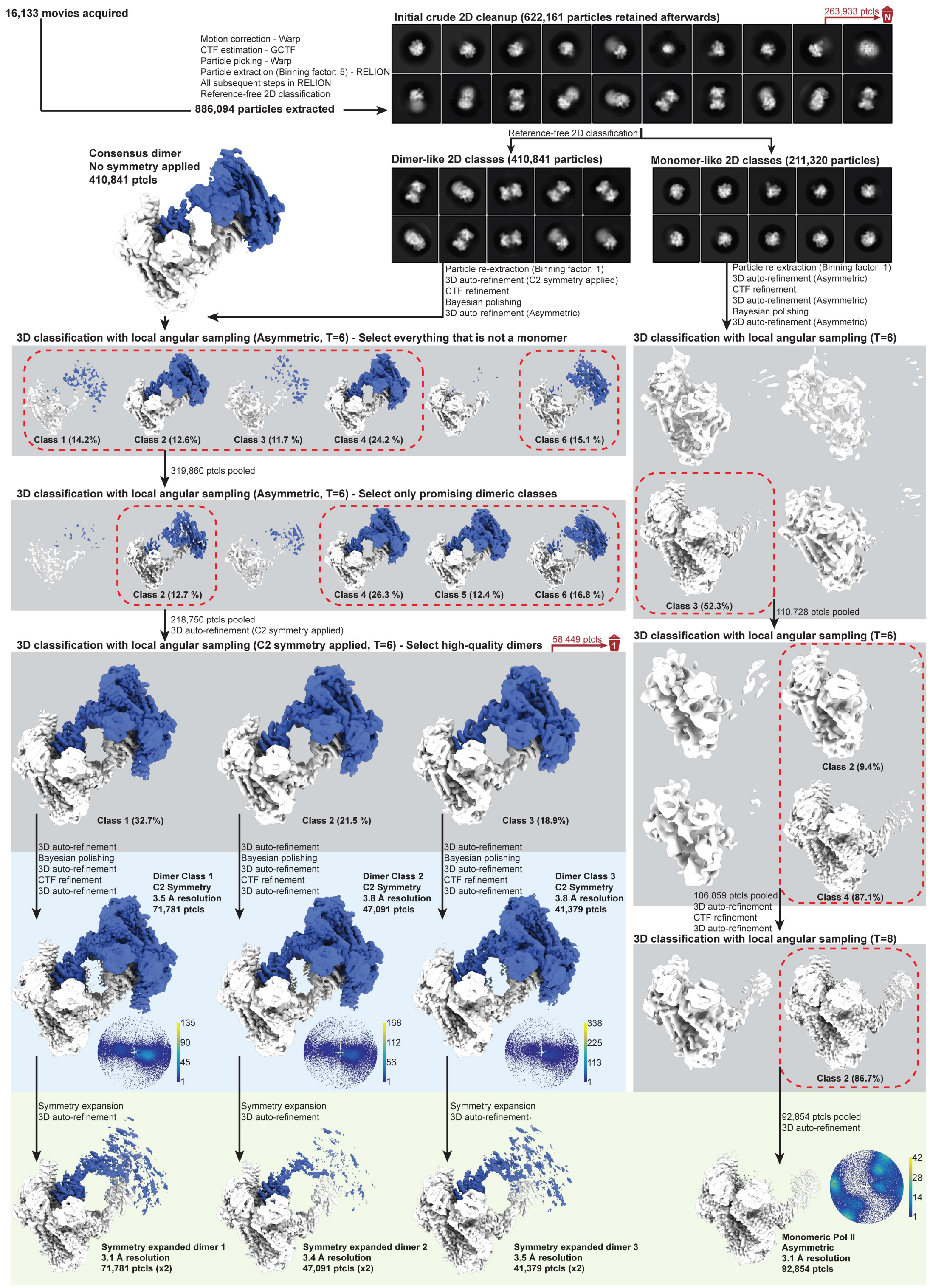

Supplementary Figure 2. Cryo-EM processing flowchart showing. Classes arising from a single 3D classification are grouped with a grey background, and classes that were pooled for the next step are circled with a red dotted line. In case where a class that was discarded that could not be shown due to space limitations, a red bin and arrow indicates the number of particles and classes (in the bin) that have been omitted for clarity. Final dimer reconstructions are shown with a sky-blue backing, the symmetry expanded version, and monomeric Pol II are shown with green backing.

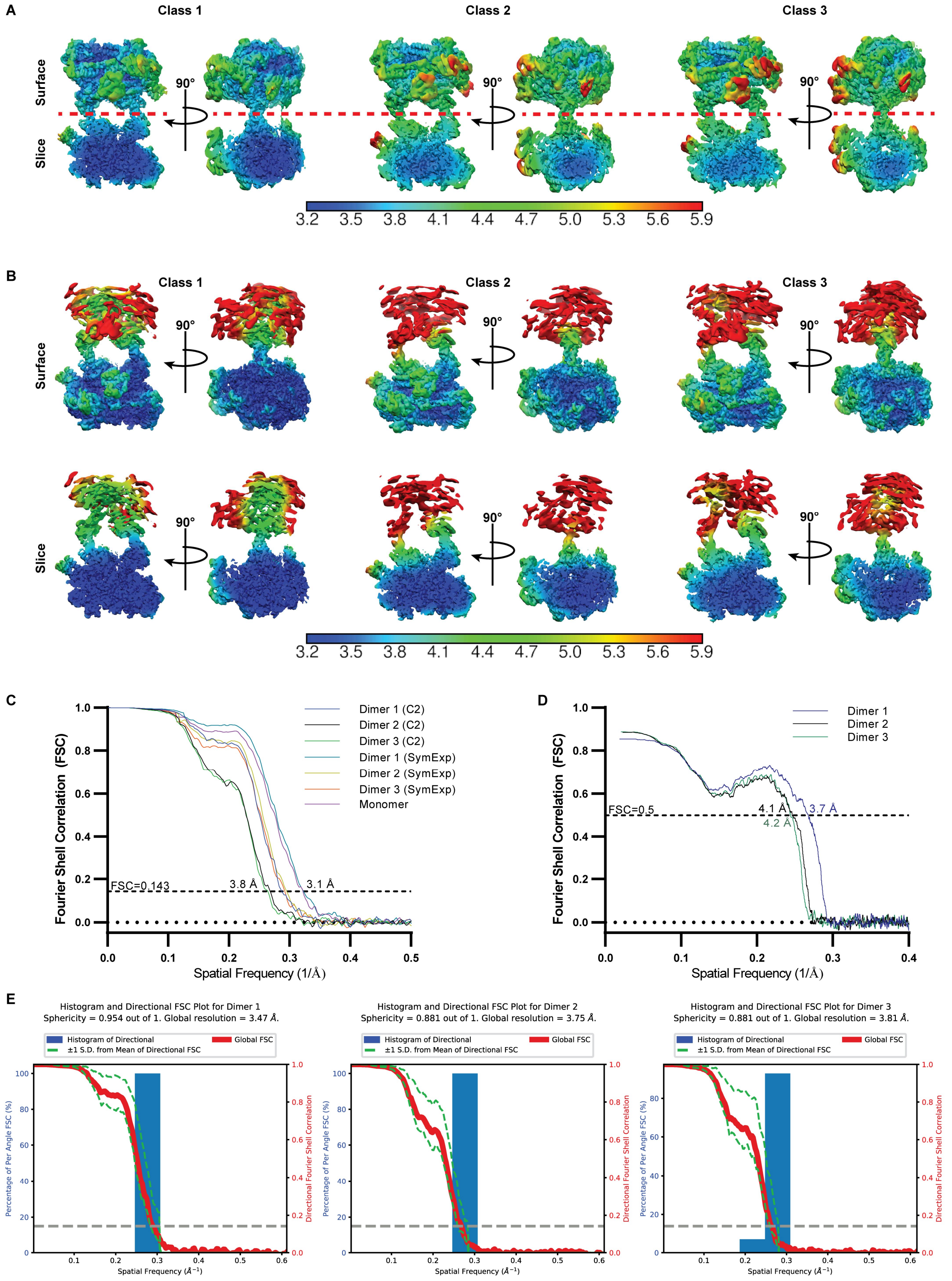

Supplementary Figure 3. Quality of cryo-EM structure. (A) Local resolution analysis of the three dimeric Pol II reconstructions with C2 symmetry applied and **(B)** for the symmetry expanded reconstruction. Top half shows the surface and the bottom half shows the central slice. **(C)** Fourier Shell Correlation of the cryo-EM maps used in this study. **(D)** Model-to-map FSCs of the three atomic models of the dimers with their corresponding cryo-EM map. **(E)** 3D-FSCs of the three dimer classes.

A

1 130
MHGGGPPSGDSACPLRTIKRVQFGVLSPDELKRMSVTEGGIKYPETTEGGRPKLGGLMDPRQGVIERTGRCQTCAGNMTECPGHFGHIELAKPVFHVGFLVKTMKVLRCVCFFCSKLLVDSNNPKIKDIL
Clamp core Clamp head

131 260
AKSKGQPKKRLTHVYDLCKGKNICEGGEEMDNKFGVEQPEGDEDLTKEKGHGGCGRYQPRIRRSGLELYAEWKHVNEDSQEKKILLSPERVHEIFKRISDEECFVLGMEPRYARPEWMIVTVLPVPPLSV
Clamp head Clamp core

261 390
RPAVVMQGSARNQDDLTHKLADIVKINNQLRRNEQNGAAAHVIAEDVKLLQFHVATMVDNELPGLPRAMQKSGRPLKSLKQRLKGKEGRVRGNLMGKRVDFSARTVITPDPNLSIDQVGVPRSIAANMTF
Clamp core Switch 2

391 520
AEIVTPFNIDRLQELVRRGNSQYPGAKYIIRDNGDRIDLRFHPKPSDLHLQTGYKVERHMCDGDIVIFNRQPTLHKMSMMGHRVRILPWSTFRLNLSVTTPYNADFDGDEMNLHLPQSLETRAEIQELAM
Active site

521 650
VPRMIVTPQSNRPVMGIVQDTLTAVRKFTKRDVFLERGEVMNLLMFLSTWDGKVPQPAILKPRPLWTGKQIFSLIIPGHINCIRTHSTHPDDEDSGPYKHISPGDTKVVVENGELIMGILCKKSLGTSAG

651 780
SLVHISYLEMGHDITRLFYSNIQTVINNWLLIEGHTIGIGDSIADSKTYQDIQNTIKKAKQDVIEVIEKAHNNELEPTPGNTLRQTFENQVNRILNDARDKTGSSAQKSLSEYNNFKSMVVSGAKGSKIN

781 910
ISQVIAVVGQQNVEGKRIPFGFKHRTLPHFIKDDYGPESRGFVENSYLAGLTPTEFFFHAMGGREGLIDTAVKTAETGYIQRRLIKSMESVMVKYDATVRNSINQVVQLRYGEDGLAGESVEFQNLATLK

911 1040
PSNKAFEKKFRFDYTNERALRRTLQEDLVKDVLSNAHIQNELEREFERMREDREVLRVIFPTGDSKVVLPCNLLRMIWNAQKIFHINPRLPSDLHPIKVVEGVKELSKKLVIVNGDDPLSRQAQENATLL

1041 1170
FNIHLRSTLCSRRMAEEFRLSGEAFDWLLGEIESKFNQAIAHPGEMVGALAAQSLGEPATQMTLNTFHYAGVSAKNVTLGVPRLKELINISKKPKTPSLTVFLLGQSARDAERAKDILCRLEHTTLRKVT

1171 1300
ANTAIYYDPNPQSTVVAEDQEWVNVYYEMPDFDVARISPWLLRVELDRKHMTDRKLTMEQIAEKINAGFGDDLNCIFNDDNAEKLVLRIRIMNSDENKMQEEEEVVDKMDDDVFLRCIESNMLTDMTLQG

1301 1430
IEQISKVYMHLPQTDNKKKIIITEDGEFKALQEWILETDGVSLMRVLSEKDVDPVRTTSNDIVEIFTVLGIEAVRKALERELYHVISFDGSYVNYRHLALLCDTMTCRGHLMAITRHGVNRQDTGPLMKC
Switch 1

1431 1560
SFEETVDVLMEAAAHGESDPMKGVSENIMLGQLAPAGTGCFDLLLDAEKCKYGMEIPTNIPGLGAAGPTGMFFGSAPSPMGGISPAMTPWNQGATPAYGAWSPSVGSGMTPGAAGFSPSAASDASGFSPG
Switch 1 Switch 5 'Acidic region'

1561 1690
YSPAWSPTPGSPGSPGPSSPYIPSPGGAMSPSYSPTSPAYEPRSPGGYTPQSPSYSPTSPSYSPTSPSYSPTSPNYSPTSPSYSPTSPSYSPTSPSYSPTSPSYSPTSPSYSPTSPSYSPTSPSYSPTSP
CTD heptapeptide repeats

1691 1820
SYSPTSPSYSPTSPSYSPTSPSYSPTSPSYSPTSPSYSPTSPSYSPTSPSYSPTSPSYSPTSPNYSPTSPNYTPTSPSYSPTSPSYSPTSPNYTPTSPNYSPTSPSYSPTSPSYSPTSPSYSPSSPRYTPQSPTYTP

1821 1950
SSPSYSPSSPSYSPTSPKYTPTSPSYSPSSPEYTPTSPKYSPTSPKYSPTSPKYSPTSPTYSPTTPKYSPTSPTYSPTSPVYTPTSPKYSPTSPTYSPTSPKYSPTSPTYSPTSPKGSTYSPTSPGYSPT

1951 1970
SPTYSLTSPAISPDDSDEEN

B

1 130
MYDADEDMQYDEDDDEITPDLWQEACWIVISSYFDEKGLVRQQLDSFDEFIQMSVQRIVEDAPPIDLQAEAQHASGEVEEPPRYLLKFEQIYLSKPTHWERDGAPSPMMPNEARLRNLTYSAPLYVDITK

131 260
TVIKEGEEQLQTQHQKTFIGKIPIMLRSTYCLLNGLTDRDLCELNECPLDPGGYFIINGSEKVLIAQEKMATNTVYVFAKKDSKYAYTGECRSCLENSSRPTSTIWVSMLARGGQGAKKSAIGQRIVATL

261 390
PYIKQEVPIIIVFRALGFVSDRDILEHIIYDFEDPEMMEMVKPSLDEAFVIQEQNVALNFIGSRGAKPGVTKEKRIKYAKEVLQKEMLPHVGVSDFCETKKAYFLGYMVHRLLLAALGRRELDDRDHYGN

391 520
KRLDLAGPLLAFLFRGMFKNLLKEVRIYAQKFIDRGKDFNLELAIKTRIISDGLKYSLATGNWGDQKKAHQARAGVSQVLNRLTFASTLSHLRRLNSPIGRDGKLAKPRQLHNTLWGMVCPAETPEGHAV

521 650
GLVKNLALMAYISVGSQPSPILEFLEEWSMENLEEISPAAIADATKIFVNGCWVGIHKDPEQLMNTLRKLRRQMDIIVSEVSMIRDIREREIRIYTDAGRICRPLLIVEKQKLLLKKRHIDQLKEREYNN

651 780
YSWQDLVASGVVEYIDTLEEETVMLAMTPDDLQEKEVAYCSTYTHCEIHPSMILGVCASIIPFPDHNQSPRNTYQSAMGKQAMGVYITNFHVRMDTLAHVLYYPQKPLVTTRSMEYLRFRELPAGINSIV

781 910
AIASYTGYNQEDSVIMNRSAVDRGFFRSVFYRSYKEQESKKGFDQEEVFEKPTRETCQGMRHAIYDKLDDDGLIAPGVRVSGDDVIIGKTVTLPENEDELEGTNRRYTKRDCSTFLRTSETGIVDQVMVT
Flap loop

911 1040
LNQEGYKFCKIRVRSVRIPQIGDKFASRHGQKGTCGIQYRQEDMPFTCEGITPDIIINPHAIPSRMTIGHLIECLQGKVSANKGEIGDATPFNDAVNVQKISNLLSDYGYHLRGNEVLYNGFTGRKITSQ

1041 1170
IFIGPTYYQRLKHMVDDKIHSRARGPIQILNRQPMEGRSRDGGLRFGEMERDCQIAHGAAQFLRERLFEASDPYQVHVCNLCGIMAIANTRTHTYECRGCRNKTQISLVRMPYACKLLFQELMSMSIAPR
Switch 3 Anchor Switch 4 Clamp

1171
MMSV

Supplementary Figure 4. Regions of disorder within RPB1 and RPB2. Primary sequences of **(A)** RPB1 and **(B)** RPB2 are displayed where the segments that were not built due to poor cryo-EM density are highlighted in red. Specific regions of interest within the disordered segments are annotated as defined in (13).

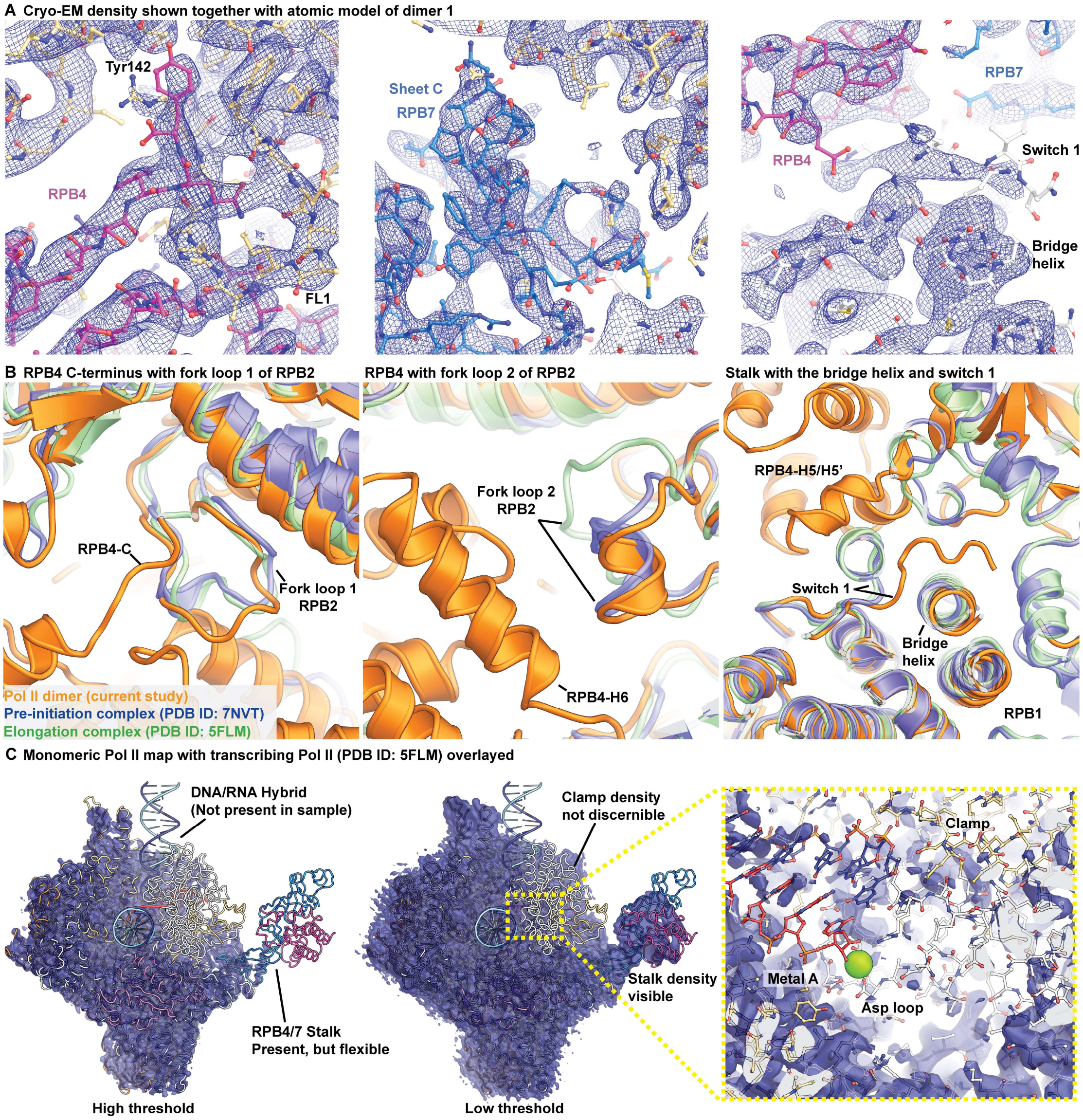

Supplementary Figure 5. Cryo-EM density and structural comparisons. **(A)** Cryo-EM density displayed together with the atomic model for dimer class 1. The three main interfaces as defined in Figure 2 are shown. **(B)** Comparison between the structure of the Pol II dimer (orange) and the pre-initiation complex (PIC, blue, PDB ID: 7NVT) or the elongation complex (EC, green, PDB ID: 5FLM). **(C)** The cryo-EM reconstruction of monomeric Pol II within our dataset shows that clamp mobility is not due to dimerization. Even in the monomeric Pol II the density for the clamp is not discernible, and a view into the active centre region shows that the density for key elements such as the aspartate loop (Asp loop) and Metal A are absent.

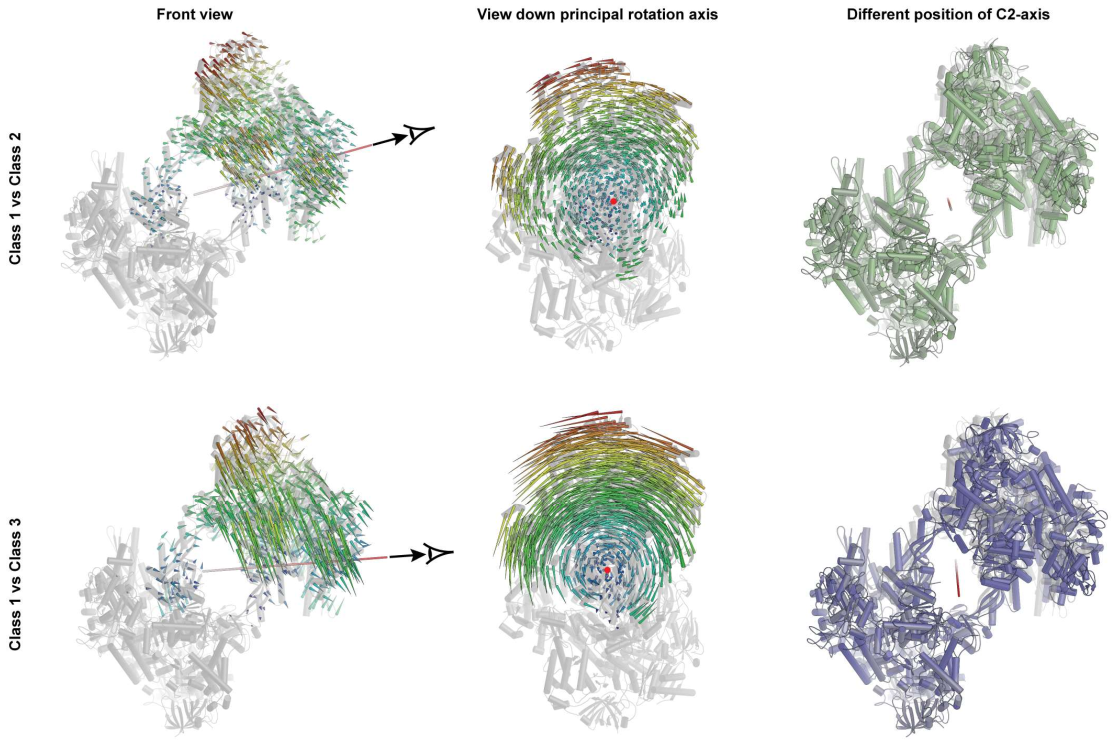

Supplementary Figure 6. Dimer flexibility. Overlay of the three classes of Pol II obtained in this study. The composition and the mode of dimerization is unchanged, but the relative position of the two copies is slightly changed, leading to a slight change in the two-fold rotation axis.

Supplementary Movie 1. Overview of the Pol II dimer with the cryo-EM density

Supplementary Movie 2. Morph between the three Pol II dimer classes from two views
